# Nanotechnology Powered CRISPR-Cas Systems for Point of Care Diagnosis and Therapeutic

**DOI:** 10.34133/2022/9810237

**Published:** 2022-09-08

**Authors:** Chen-zhong Li, Tony Y. Hu

**Affiliations:** Center for Cellular and Molecular Diagnostics, Department of Biochemistry and Molecular Biology, Tulane University School of Medicine, 1324 Tulane Ave. New Orleans, LA 70112, USA

Inspired by the successful application of Cas9 gene editing for “genome surgery,” we are looking back at what has been accomplished in the ten years of CRISPR editing. Intense effort is now focused on how to utilize the unique functions of CRISPR-Cas systems for molecular diagnostics and the next generation of therapeutic tools. CRISPR-Cas systems have two major advantages for gene editing that render them attractive for these new applications, specifically the ability to easily control their target recognition specificity and to couple this specificity to a targeted or secondary cleavage activity. However, despite the great potential of CRISPR/Cas for both gene editing and diagnosis, its medical applications have yet to be fully realized due factors that limit their delivery or utility for point-of-care (POC) applications. In this editorial note, we discuss how nanotechnology can address challenges associated with new CRISPR applications and what nanotechnology-specific advances are needed to circumvent remaining barriers that limit the development or use of new CRISPR/CAS-based genome editing and diagnostic approaches.

CRISPR/Cas-mediated gene editing approaches require a delivery system that efficiently delivers their reagents to target cells, but conventional delivery approaches (e.g., viral vectors and targeted electroporation) used in other gene editing methods can have limitations when applied to deliver CRISPR/Cas reagents [1]. For example, Cas9 is a larger ribonucleoprotein (RNP, 160 kDa, 10 nm diameter) than traditional nucleic acid vectors and contains a substantial negative charge from its crRNA component. It a significant challenge to package this RNP directly into a delivery platform that can protect it from denaturation and degradation during both carrier formulation and *in vivo* delivery. Nanoparticle platforms exhibit strong potential to address issues associated with CRISPR reagent delivery, but several nanotechnology-specific considerations are needed to realize the potential of these delivery systems. For example, some nanomaterial-mediated delivery platforms are based on chemisorption of the polynucleotide cargo to the nanoparticle. Thus, at the most basic level, cationic lipid-based nanoparticles (NPs) can be used to encapsulate CRISPR components for cell delivery through charge interactions. However, this approach can have potential toxic effects and nonspecific NP uptake that can produce off-target effects and limit bioavailability to desired cell target cells. This can be remedied using lipid-based NPs that incorporate targeting molecules, including NPs derived from the plasma membranes of cells that express, or can be engineered to express, factors that target the cell of interest. Such membrane-derived NPs can also exhibit high biocompatibility to minimize immune reactions that can limit the effect of gene editing approaches.

Aside from their these encapsulation and biocompatibility properties, NP delivery systems also needs to overcome physical barriers and direct carry CRISPR/Cas9 components to targeted cells and tissues for accurate and efficient genome editing. Synthetic NP designs should thus accommodate reactive chemical groups on their outer surfaces to permit modifications with factors that can be used to enhance their *in vivo* stability, targeting, and cell entry characteristics. NP platforms could also be employed to improve targeted release of CRISPR RNPs to better control spatiotemporal regulation of gene editing activity. Promising NP-based vectors employed for efficient delivery of CRISPR-Cas RNPs to date include liposomal/lipid-based NPs, polymeric NPs/polyplexes, gold NPs, synthetic and engineered exosomes, mesoporous silica nanoparticles (MSNPs), metal–organic frameworks (MOFs), and multiple cationic NPs (e.g., polyamidoamine (PAMAM)-based dendrimers, polyethyleneimine (PEI), and chitosan NPs) [2]. These nanotechnology-based smart delivery systems thus have the potential to significantly improve current gene editing approaches by increasing targeted delivery to reduce off-target effects and immune reactions that can complicate the design and application of gene therapy approaches.

Integrating nanotechnology with CRISPR assay approaches also provides a powerful new means for the fast, specific, and ultrasensitive detection of protein biomarkers, whole cells, and small molecules, in addition to nucleic acid targets [3]. The detection system can employ a variety of readouts, including fluorescent, colorimetric, plasmonic, and electrochemical signals, but the most common approach used relies on the detection of fluorescence produced upon CRISPR-mediated cleavage of RNA or DNA reporters that carry a fluorophore and a quencher at their opposing termini. The incorporation of nanomaterials as signal readouts can however enhance detection sensitivity or reduce the need for special equipment for signal detection. Furthermore, nanomaterials such as graphene, MXene nanosheets, or nanomaterial combinations can also be incorporated to CRISPR/Cas technology acting as highly sensitive electrodes to detect electrochemical signals produced by CRISPR cleavage of the probes labeled by electrochemical active tags, for use in point-of-care (POC) applications. New POC tests are needed to improve healthcare in diagnostic settings with limited resources, and this demand has become more pressing since the COVID-19 pandemic, as diversion of resources to meet COVID-19 testing demands has led to substantial deficits in the capacity to monitor other major diseases and health conditions. Given the recent global spread of digital infrastructure, wireless networks, and mobile devices, CRISPR/Cas POC diagnostic platform should ideally incorporate detection systems that meet the World Health Organization's (WHO's) REASSURED criteria: Real-time connectivity, Ease of specimen collection, Affordable, Sensitive, Specific, User-friendly, Rapid and robust, Equipment-free or simple, and Deliverable to end-users [4].

New assay designs and device nanofabrication approaches are required to develop applications that simplify sample preparation, target preamplification, and provide multiplexed diagnostic capacity [5]. The specific high-sensitivity enzymatic reporter unlocking (SHERLOCK) assay recently approved by the FDA for SARS-CoV-2 detection combines a lateral-flow strip readout that exhibits attomolar target sensitivity. Integrating of CRISPR/Cas assays with microfluidic systems has also introduced new opportunities for target detection and reporting on surfaces integrated into wearable devices and cellphone-based assay readers. Compared to solution-based CRISPR/Cas detection methods, such surface-based sensing approaches benefit from high local concentrations of target biomarkers on the assay surface and may permit continuous and robust monitoring of diagnostic targets at home and routing during activity. Integrating machine learning, bioinformatic algorithms, and data-sharing into such devices holds promise for more effective patient-centric testing and remote data access for improved disease detection and treatment monitoring and to mitigate the risk of disease transmission.

CRISPR/Cas diagnostic platforms still have limitations, however, including a need for target amplification, short-term shelf life, nanomaterial degradation in biospecimens, and the potential complexity of the design and fabrication of their sensing systems. The incorporation of nanomaterials into CRISPR/Cas assay systems and devices has been limited thus far but there is significant potential for such applications to enhance the diagnostic performance of CRISPR/Cas assays.
